# Prediction of leisure-time walking: an integration of social cognitive, perceived environmental, and personality factors

**DOI:** 10.1186/1479-5868-4-51

**Published:** 2007-10-31

**Authors:** Ryan E Rhodes, Kerry S Courneya, Chris M Blanchard, Ronald C Plotnikoff

**Affiliations:** 1 School of Exercise Science, Health and Physical Education, University of Victoria, Victoria, Canada; 2 Faculty of Physical Education, University of Alberta, Edmonton, Canada; 3 Faculty of Medicine, Dalhousie University, Halifax, Canada

## Abstract

**Background:**

Walking is the primary focus of population-based physical activity initiatives but a theoretical understanding of this behaviour is still elusive. The purpose of this study was to integrate personality, the perceived environment, and planning into a theory of planned behaviour (TPB) framework to predict leisure-time walking.

**Methods:**

Participants were a random sample (N = 358) of Canadian adults who completed measures of the TPB, planning, perceived neighbourhood environment, and personality at Time 1 and self-reported walking behaviour two months later.

**Results:**

Analyses using structural equation modelling provided evidence that leisure-time walking is largely predicted by intention (standardized effect = .42) with an additional independent contribution from proximity to neighbourhood retail shops (standardized effect = .18). Intention, in turn, was predicted by attitudes toward walking and perceived behavioural control. Effects of perceived neighbourhood aesthetics and walking infrastructure on walking were mediated through attitudes and intention. Moderated regression analysis showed that the intention-walking relationship was moderated by conscientiousness and proximity to neighbourhood recreation facilities but not planning.

**Conclusion:**

Overall, walking behaviour is theoretically complex but may best be addressed at a population level by facilitating strong intentions in a receptive environment even though individual differences may persist.

## Background

Physical activity (PA) promotion is a public health priority. PA itself, however, is a collection of behaviours and the promotion of specific modalities may be important. Walking behaviour has received recent attention based on its physical [1,2] and psychological [3] health benefits and its high preference in terms of activity choice among adults [4]. These aspects suggest that promotion of regular walking should be the primary focus of population-based PA promotion efforts.

PA promotion should be theory-based [5,6]. One theory that has been extensively validated in the PA domain is Ajzen's [7] theory of planned behaviour (TPB) [8]. The TPB proposes that the final pathway to behaviour is intention: one's overall motivation to perform the behaviour. Intentions, however, can only be carried out in a receptive environment over which the person has control. An objective measure of control is elusive, but perceived behavioural control (PBC) is often a good proxy measure of actual control [7,9]. Intention, in turn is thought to be influenced by affective (e.g., evaluation of the enjoyment of performing the behaviour) and instrumental (e.g., evaluation of the benefit of performing the behaviour) attitudes, subjective norm (e.g., evaluation of the perceived approval from others to perform the behaviour) and PBC (i.e., perception of capability to perform the behaviour when motivation is assumed [10]).

Evaluation of the TPB for predicting walking has been scant. Three studies [11-13] have evaluated the TPB and walking and these show relatively similar results to general PA meta-analysis in terms of intention-behaviour relations [8]. Rhodes et al. [11], however, showed that PBC was not related to walking whereas the findings of Eves et al. [12] and Scott et al. [13] suggested that attitude was not a predictor of walking intention or behaviour. The difference in findings may be from geographical variation (Western Canada vs. U.K.), measurement differences (general walking vs. leisure-time walking), or sampling fluctuations. Clearly, more work on the TPB applied to walking is needed.

An understanding of walking may also benefit from adding breadth and depth to the TPB model and approaches to integrating PA correlates are advocated [14,15]. TPB proposes that variables external to the model should be mediated via its constructs of attitude, subjective norm, and PBC when considering their respective associations with behaviour [7]. In addition, external factors may moderate the TPB model. Of particular interest in this regard is the intention-behaviour relationship [16]. An understanding of moderators of the intention-behaviour link is very important because a majority of the population reports positive PA intentions but discordant actual PA [17]. Three factors at different theoretical levels of abstraction that have all shown application to augment the TPB are the perceived environment, personality, and action planning/implementation intentions.

Environmental factors are often a focus in walking related research [18]. This literature has some mixed findings, but almost all studies have converged on the importance of proximity to amenities (e.g., markets, retail stores) and the perceived aesthetics of the neighbourhood (e.g., attractive scenery, well-maintained homes) as the key correlates of walking [18-22]. The integration of these environmental factors with social cognitive constructs, however, is limited at present [11,23-25]. The only study to include the perceived environment within a TPB model to predict walking found that these perceived environment factors associated with walking (i.e., neighbourhood aesthetics, proximity to retail) were mediated by attitudes [11]. Therefore, the tenet of the TPB in terms of mediation of behaviour of "outside" factors was supported. Also, perceived proximity to recreation moderated intention-walking relations, with those perceiving a closer proximity showing a larger intention-walking relationship than those who reported being farther away from recreation infrastructure. These results suggest that the environment may affect walking behaviour through attitudes and moderate the intention behaviour gap. Still, the finding requires replication.

Another factor that has received recent attention in the PA domain with the TPB is personality [26]. Personality is generally defined as stable individual differences in thoughts, feelings, and actions [27] and a recent meta-analytic review of PA and personality found that extraversion (tendency to show positive disposition, be sociable and lively, etc.), and conscientiousness (tendency to be orderly, self-disciplined, etc.) are positive correlates of PA while neuroticism (tendency to show negative disposition, self-reproach, etc.) is negatively correlated [28]. Research integrating the TPB with personality and PA has generally found a failure of the TPB to fully mediate extraversion or conscientiousness [28]. Those study authors have suggested that the stability of personality may augment the more transient predictive ability of social cognitions on PA across time. Further, conscientiousness may moderate the intention-behaviour relationship (conscientious individuals displaying stronger intention-PA associations than their less conscientious counterparts) [28,29]. Still, almost no research has evaluated personality and walking. Early work by Howard et al. [30] found that extraversion was correlated with more vigorous intensity forms of PA and was not related to walking for exercise. Additional research focusing on walking and personality, and a test of whether conscientiousness moderates the intention-walking relationship is warranted.

Finally, a construct receiving considerable attention and support in the health behaviour domain is planning [31-34]. It has been suggested by Gollwitzer and colleagues [31,35] that models like the TPB are motivational in nature, but specific volitional plans (e.g., if...then, when, why, where, how) may be necessary to translate intentions into behaviour. Thus, planning may add depth to the TPB framework by acting as a mediator between intention and behaviour or moderate intention-behaviour relations. Research on planning and PA in the TPB has shown results in support of this theorizing [e.g., [32,33,36]], but not all studies have shown this distinction [33,34]. Research is needed to evaluate the addition of a planning construct when understanding regular walking.

Therefore, the purpose of this study was to incorporate personality, the perceived environment, and planning into a TPB framework to predict leisure-time walking. Based on prior research, it was hypothesized that the perceived environment, most notably neighbourhood aesthetics and proximity to retail shops, would be correlates of walking but mediated through attitudes about walking (and subsequent intention to walk). Personality constructs of extraversion, neuroticism, and conscientiousness were expected to be unrelated to walking based on prior work [30] and thus not of utility in an integrated TPB model, and planning was hypothesized to act as a mediator of intention and walking relations [33]. Further, based on prior work [11,29,33,37,38], we hypothesized that the perceived proximity to recreation infrastructure, planning, and conscientiousness would moderate the intention-walking relationship.

## Method

### Participants and design

Participants for this study were residents of British Columbia (BC), Canada aged 18 or greater. A random sample of 1500 addresses within BC was obtained from Dominion Directories (SuperPages Telephone Company). In February 2005, questionnaires approved by the University of Victoria's Human Research Ethics Board were mailed to the 1500 potential participants. Of the original 1500 questionnaires, 222 envelopes were returned unopened because the resident had moved (n = 208) or was recently deceased (n = 14), and 232 questionnaires were returned completed. Of the possible 1046 remaining participants, a second mailing of a post card reminder and questionnaire [39,40] was sent out two weeks later in which an additional 126 questionnaires were returned. Thus, a total of 358 participants (28% of eligible participants) completed and returned the questionnaire. A second follow-up mail-out to these 358 participants was conducted two months after receipt of their completed survey. Of these participants, 203 individuals completed and sent back the follow-up questionnaire comprised of a measure of walking over the past two months (57% follow-up rate).

Of the 358 participants at baseline, 51% were males and 49% were females with a mean age of 57.0 (SD = 14.6) and 50.6 (SD = 16.9) respectively. Respondents reported themselves as well-educated; 49.2% had at least a Bachelors degree or certificate, which is above the 35% reported during the Census of 2001 [41]. Of those reporting race (n = 334), 80.9% were Caucasian which is very close to the BC census of 78.6% [41]. Other participant characteristics were similar to the general population of British Columbia. Only 2.3% were unemployed, with 35.8% being retired, 1.1% attending College or University, 54.3% employed, and 2.5% on leave. Annual family income showed 60.7% had a household income over 40,000 CDN per year, which is the BC median [41]. Finally, 65.3% of participants were married/common-law, 22.9% were separated/widowed, and 11.9% reported themselves as single.

For health indicators, 10% of the sample were smokers, 23% reported having high cholesterol, 2.9% had a stroke, 5.1% reported having a heart attack in the past, 24.5% had high blood pressure, 9.4% had diabetes (64.5% type 2), 8.6% were cancer survivors, and the mean BMI was 25.71 (SD = 4.59). In terms of physical activity, 17% were aware of Health Canada's Guide to Healthy Active Living, which is similar to prior research in Canada [42]. Finally, using the Godin Leisure-Time Questionnaire [43] to measure physical activity, 48.8% were meeting Canada's physical activity guidelines [44] which is lower than the 58% reported for the province [4]. These data have also been previously reported in a study focused on the TPB belief-level constructs [45].

### Instruments

Perceived environment characteristics were based on a set of items from the Neighbourhood Environment Walkability Scale (NEWS)[46,47] and the International Physical Activity Prevalence Study Environmental Survey Module (IPAPSEM)[48] that have been used to predict walking in prior research [11]. Measurement of the perceived environment is relatively unstandardized at present, but these measures and previous research [18] highlight proximity to retail or recreation, aesthetics, crime, traffic, and walking infrastructure quality as key characteristics. We decided to follow the IPAPSEM approach of clear (i.e., high face validity) single item indicators for each characteristic. This decision was predicated on the overall length of the NEWS in comparison to the IPAPSEM, particularly in consideration of the other measures included in this survey, but with some preference for NEWS items. Thus, proximity was assessed with the items: (1) "Many shops, stores, markets or other places to buy things I need are within easy walking distance of my residence" (retail), and (2) "My neighbourhood has several free or low cost recreation facilities, such as parks, walking trails, bike paths, and recreation centers" (recreation). Walking infrastructure quality was measured by the item: "There are well-maintained sidewalks on most of the streets in my neighbourhood," and neighbourhood aesthetics was measured using the item: "There are many attractive natural sights in my neighbourhood (such as landscaping, views...)." Finally, traffic was measured with the item: "It feels unsafe to walk along the streets in my neighbourhood because there is so much traffic," and crime was measured with the item: "There is a high crime rate in my neighbourhood." All items were answered using a four-point scale from strongly disagree (1) to strongly agree (4) which is similar between the NEWS and IPAPSEM measures.

Personality traits of extraversion, neuroticism, and conscientiousness were measured using unipolar trait markers originally developed and validated by Golberg [49] and further cross-validated by Saucier and Ostendorf (1999). These phenotypic trait measures have shown identical relationships with PA when compared to genotypic measures such as the NEO-FFI or EPI [28]. Participants were asked to describe themselves as accurately as possible as they are typically or generally as compared with persons they know of the same gender and roughly the same age. Six unipolar markers were used for each trait and rated on 5 point scales from 1 (extremely inaccurate) to 5 (extremely accurate). Internal consistencies were acceptable for the extraversion (α = .70), conscientiousness (α = .72) and neuroticism (α = .74) measures.

Walking was measured using a variant of the Godin Leisure Time Exercise Questionnaire (GLTEQ) [43,50,51]. This measure has been used in prior walking studies [3,11,52]. The decision to use this adapted GLTEQ was also made because of our inclusion of general PA in the survey. It made sense to have two very analogous measures of both PA and walking because similar framing of measures eases response burden and reduces error [53]. Participants were asked to recall their average weekly walking during their free time over the past two months. The GLTEQ contains three open-ended PA questions pertaining to the average frequency of mild, moderate, and strenuous physical activities (with examples of each) during free time in a typical week. For walking, mild, moderate and strenuous physical activities were changed to mild (Minimal effort, no perspiration, a casual walk), moderate (Not exhausting, light perspiration, a good brisk pace) and strenuous (Heart beats rapidly, sweating, as fast as you could walk) walking respectively. We also modified the GLTEQ to include an open assessment of average duration. Frequencies of strenuous (20 minutes+), moderate (30 minutes+), and mild (60 minutes+) were aggregated to produce a total walking frequency score that corresponds to Health Canada's current PA recommendations [44].

TPB constructs were measured using 7-point Likert type questions. For the TPB questions, regular walking was defined as "walking for at least 30 minutes, at least 4 times or more per week during your free time." This definition is based Health Canada's recommended minimum guideline for physical activity.

Attitude towards regular leisure-time walking was measured using three items that tap the instrumental (i.e., useful-useless, wise-unwise, beneficial-harmful) component, and three items that tap the affective (enjoyable-unenjoyable, pleasant-unpleasant, exciting-boring) component. The response format was a series of 7-point scales (1,7 = extremely, 2,6 = moderately, 3,5 = slightly) and the phrase that preceded these scales was "For me, regular leisure-time walking over the next 2 months would be...". Cronbach's alpha coefficients of internal consistency were 0.78 for instrumental attitude and 0.76 for affective attitude.

Subjective norm was measured by combining two items assessing the injunctive component of subjective norm and one item that tapped the descriptive component. The items were: (1) "Most people who are important to me want me to engage in leisure-time walking over the next 2 months," (2) "Most people whose opinions I value would approve of me engaging in leisure-time walking over the next 2 months," and (3) "Most people who are important to me will engage in regular leisure-time walking themselves over the next 2 months" The combination of these components (injunctive and descriptive) was based on recommendations of Ajzen [9] and the results of Rhodes and colleagues [54,55]. Cronbach's alpha coefficient of internal consistency was 0.72.

Perceived behavioural control was measured by three items that have been previously recommended [9,56]. The items were: (1) "In the next 2 months, I have complete personal control over leisure-time walking if I really wanted to do so," (2) Engaging in leisure-time walking is mostly up to me in the next 2 months if I wanted to do so," and (3) Engaging in leisure-time walking over the next 2 months if I wanted to do so would be...". The first two items were scored on a 7-point scale ranging from 1 (strongly disagree) to 7 (strongly agree), while the third item was scored from 1 (extremely difficult) to 7 (extremely easy). The items were standardized before the aggregate measure was created (α = .86).

Intention and planning were measured with attention to reducing potential measurement confounds. Based on the recommendations of Rhodes et al. [34], intention was measured without the use of "intend" and "plan" items and instead by items that reflect motivation. The two items were: (1) "I am motivated to engage in regular leisure-time walking over the next 2 months," from 1 (extremely unmotivated) to 7 (extremely motivated), and (2) "I am determined to engage in regular leisure-time walking over the next 2 months," from 1 (extremely undetermined) to 7 (extremely determined). Internal consistency was α = 0.92. Planning was measured using items created by Rise et al. [36] and further validated by Rhodes et al. [34]. These items were: (1) "I have made plans concerning "when" I am going to engage in leisure-time walking over the next 2 months," (2) "I have made plans concerning "where" I am going to engage in leisure-time walking over the next 2 months," (3) "I have made plans concerning "what" kind of walking (e.g., brisk exercise, casual social, etc.) I am going to engage in over the next 2 months" and (4) "I have made plans concerning "how" I am going to get to a place to engage in leisure-time walking over the next 2 months." These items were scored from 1 (strongly disagree) to 7 (strongly agree) and internal consistency was acceptable (α = 0.93).

### Analyses

#### Preliminary analyses

Although complete data were available at baseline, 155 participants did not complete the two month walking assessment. To determine the pattern of missingness surrounding walking, a dummy variable was created (0 = walking data absent; 1 = walking data present). Next, this variable was compared on the baseline walking, demographic and TPB variables via zero-order correlations and χ^2 ^analyses. Results showed that walking missingness was significantly (p < .05) related to being less educated, living alone, and reporting a lower income. Therefore, the data were not missing completely at random. Still, it was assumed that the data were missing at random because the probability of missing a walking data point was not related to its particular value (i.e. baseline walking), but was dependent upon these other variables [57]. Missing values were thus imputed using the expectation maximization algorithm [57] in LISREL 8.8. Bivariate correlation and regression results were compared between the imputed values and values using listwise deletion of missing data to assess the effect of the missing data procedure. Results using Hotelling's t for dependent correlations were not significantly different (p < .05), suggesting that the procedure had the intended effect of increasing power (through the inclusion of the larger N) but not changing the results.

#### Analysis plan

Bivariate correlations of perceived environment variables, personality, and TPB constructs with walking were evaluated. To create parsimony in the integrated path model, only significant (p < .05) bivariate correlations between personality/perceived environment and walking were integrated with the TPB to predict walking. This data reduction step was considered acceptable because a basic bivariate correlation with the dependent variable of interest (i.e., walking) is necessary to even establish the potential for mediation [58].

Analyses of the integrated model used structural equation modeling [59] with maximum likelihood estimation and a covariance matrix. Specifically, the environmental/personality variables were modeled as antecedents of the TPB model, which was subsequently used to predict walking. The planning variable was modeled as the proximal predictor of walking with intention and PBC as its respective antecedents [34]. The first indicator of each latent variable was fixed to 1.0 in order to create a metric scale. Single indicators (i.e., environment variables, walking) were fixed to 0 error, which is commensurate with ordinary least squares regression analyses. The environment/personality variables were freed to correlate, and the structural disturbance terms (residual variance) among TPB variables of affective attitude, instrumental attitude, subjective norm, and PBC were also freed to correlate amongst each other as per the tenets of the TPB [7]. Mediation was evaluated by comparing this model to a model where the direct paths of the environmental/personality variables were freed upon walking in conjunction with assessment of indirect effects. A nonsignificant (p > .05) χ^2 ^supports mediation [58].

To evaluate whether proximity to recreation, planning, and conscientiousness variables moderated intention and walking relations, we mean-centered all variables [60] and followed the procedure suggested by Cohen & Cohen [61] using ordinary least squares multiple regression. All hypothesized variables were analyzed simultaneously. Specifically, intention, planning, conscientiousness, and proximity to recreation variables were entered into the regression equation first, and interaction terms were then entered into the regression equation in a second block. Finally, interpretation of significant interaction effects used Aiken & West's [60] suggested procedure of slope analysis. Type one error was set at p < .05.

## Results

Bivariate correlations and descriptives for all variables of interest with walking can be found in Table 1. All social cognitive constructs correlated with walking and the results were in the medium effect size range [62]. By contrast, personality variables did not correlate with walking and only perceived environmental variables of proximity to retail (r = .17), infrastructure quality (r = .17), and aesthetics (r = .14) were significant correlates.

**Table 1:** Correlations of social cognition, perceived environment and personality with walking (N = 358).

Construct	M	SD	Walking
TPB Constructs			
Affective Attitude	5.48	0.92	.32**
Instrumental Attitude	6.19	0.82	.25**
Subjective Norm	5.60	1.11	.24**
PBC	5.92	1.27	.27**
Intention	5.51	1.42	.41**
Planning	5.01	1.80	.29**
			
Perceived Environment			
Proximity to Retail	2.75	1.16	.17**
Proximity to Recreation	3.37	0.86	.09
Infrastructure Quality	3.08	1.14	.17**
Neighbourhood Aesthetics	3.31	0.77	.14**
Traffic Safety	1.74	0.89	-.01
Crime	1.67	0.85	.07
			
Personality			
Extraversion	4.93	0.79	.03
Neuroticism	3.59	0.91	.03
Conscientiousness	5.33	0.90	.08

Note: * *p *< .05; ** *p *< .01. PBC = perceived behavioural control. TPB = theory of planned behaviour.

**Figure 1:**
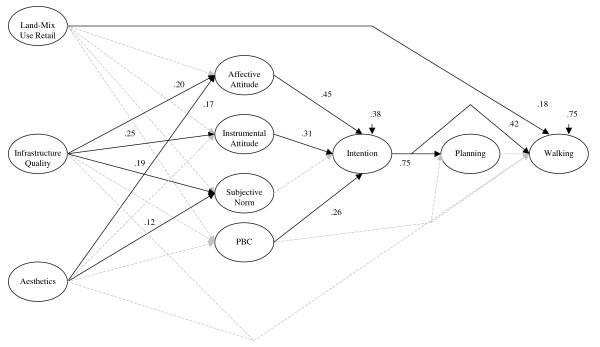
Perceived environment and theory of planned behaviour model to predict walking. Note: All effects are standardized;  = *p *< .05,  = *p *> .05.

Because a significant relationship with the dependent variable of walking is required in mediation analyses, only neighbourhood aesthetics, infrastructure quality and proximity to retail were carried forward in the subsequent analysis. This next analysis integrated these three perceived environment variables with the TPB and planning to predict walking. The model resulted in a moderate fit of the data [χ^2 ^(183) = 716.32; *p *< .01; CFI = .95; RMSEA = .08] using conventional cut-off criteria and considering the complexity and size of the model [63]. Still, freeing the direct paths for the perceived environmental variables on walking improved fit and explained an additional 2% of the variance in walking beyond the social cognitive constructs (Δχ^2 ^(3) = 18.90; p < .01). The addition of the direct paths of the environmental variables on intention, however, did not add to the overall fit (Δχ^2 ^(3) = 6.43; p > .05). The final structural model is presented in Figure 1 (covariance results among TPB constructs and environmental variables have been omitted for illustrative parsimony) and the measurement model with descriptives is presented in Table 2. The full correlation matrix used for the model can be found in Table 3.

**Table 2:** Factor loadings of selected environmental characteristics, the theory of planned behaviour and walking (n = 358).

	Mean	SD	Factor Loading	Error Variance
Perceived Environment				
Proximity to Retail	2.76	1.16	1.00	.00
Infrastructure Quality	3.08	1.14	1.00	.00
Neighbourhood Aesthetics	3.31	0.77	1.00	.00
				
Affective Attitude				
Enjoyable-unenjoyable	5.79	1.19	.87	.24
Pleasant-unpleasant	5.93	1.01	.90*	.19
Exciting-boring	4.69	1.16	.63*	.60
				
Instrumental Attitude				
Useful-useless	6.14	0.94	.79	.37
Wise-foolish	6.21	1.06	.87*	.24
Beneficial-harmful	6.22	0.91	.84*	.29
				
Subjective Norm				
Item 1	5.52	1.53	.80	.35
Item 2	6.31	1.14	.87*	.24
Item 3	4.95	1.47	.56*	.68
				
Perceived Control				
Item 1	5.99	1.51	.96	.08
Item 2	6.20	1.35	.92*	.16
Item 3	5.57	1.45	.77*	.41
				
Intention				
Item 1	5.55	1.45	.92	.16
Item 2	5.47	1.50	.96*	.08
				
Planning				
"when"	4.77	2.04	.92	.15
"where"	5.14	1.91	.95*	.10
"what"	5.15	1.85	.93*	.14
"how"	4.94	2.08	.86*	.26
				
Walking				
GLTEQ	4.27	2.09	1.00	.00

Note: All loadings reported are standardized. No t-values are available for the first loading, because it was fixed for model identification purposes. * All freed factor loadings significant *p *< .01.

**Table 3:** Correlation matrix of selected environmental characteristics, the theory of planned behaviour and walking (n = 358).

	2	3	4	5	6	7	8	9	10
1. Proximity to Retail	.58	.02	.11	.06	.08	.13	.11	.09	.17
2. Infrastructure Quality		.30	.25	.20	.21	.17	.22	.17	.17
3. Neighbourhood Aesthetics			.23	.06	.18	.08	.15	.12	.14
4. Affective Attitude				.69	.54	.11	.70	.53	.37
5. Instrumental Attitude					.71	.12	.67	.51	.32
6. Subjective Norm						.22	.55	.42	.28
7. Perceived Behavioural Control							.36	.31	.24
8. Intention								.76	.47
9. Planning									.29
10. Walking									

Overall, the measurement model suggested good measurement of the TPB constructs and planning with significant and large factor loadings. For the structural model, 25% of the variance in walking was explained. When considering the environmental variables, only the direct effect of proximity to retail was significant on walking (standardized effect = .18). This variable, however, had no relationship with the social cognitive constructs. By contrast, the significant indirect effects for infrastructure quality (standardized = .09) and neighbourhood aesthetics (standardized = .05) predicting walking through affective and instrumental attitudes and intention.

These effects, however, did not account for all covariation among TPB constructs of affective attitude, instrumental attitude, subjective norm, or PBC as correlated structural disturbance terms of affective and instrumental attitude (standardized effect = .64), affective attitude and subjective norm (standardized effect = .47), instrumental attitude and subjective norm (standardized effect = .66), and subjective norm and PBC (standardized effect = .19) were significant (*p *< .05). Further, because personality and other environmental variables (not included in the final structural equation model) may attenuate the effects of infrastructure quality and aesthetics on attitudes, we also evaluated all these variables simultaneously as having effects on affective and instrumental attitude in a sub-analysis. The attenuation effects were present, reducing standardized coefficients by approximately .05, but they did not alter the significance (p < .05) of infrastructure quality and aesthetics on attitudes. This suggests that the omission of these variables does not drastically alter the model.

The social cognitive part of the model showed that intention had the only significant independent effect (standardized effect = .42) on walking. Affective attitude (standardized effect = .19), instrumental attitude (standardized effect = .13), and PBC (standardized effect = .11), however, had significant indirect effects on walking through intention. Only intention explained planning (standardized effect = .75), but the planning construct had no subsequent contribution in the model. Affective attitude (standardized effect = .45), instrumental attitude (standardized effect = .31), and PBC (standardized effect = .26) subsequently explained 62% of the variance in intention. Subjective norm did not have an effect on intention independent of the other TPB constructs.

**Figure 2:**
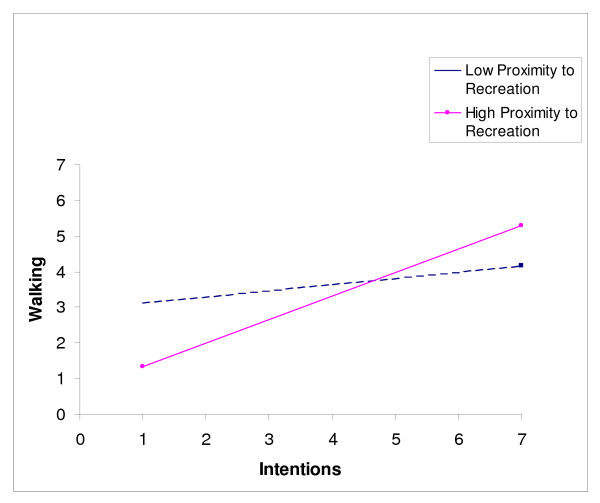
Proximity to recreation as a moderator of the intention-walking relationship.

**Figure 3:**
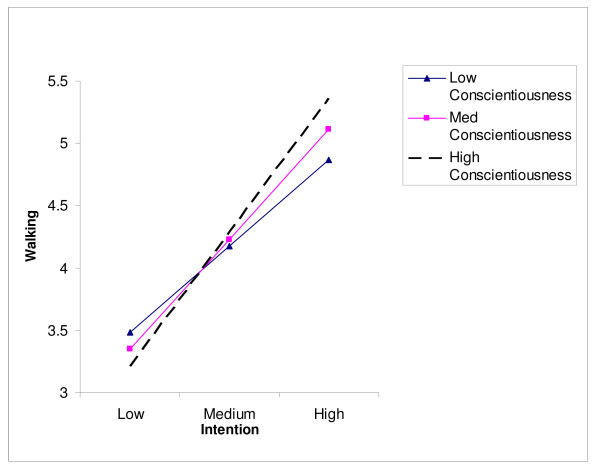
Conscientiousness as a moderator of the intention-walking relationship.

The intention-walking moderator analysis is presented in Table 4. Two of the three possible moderators of the intention-walking relationship were significant and explained an additional 2% of behaviour. Specifically, proximity to recreation and conscientiousness moderated the effect of intention on walking [F_change _(3,337) = 2.95, *p *< .05]. Specific univariate analyses also yielded the same finding with planning showing no moderator effect [F_change _(1,348) = 0.15, *p *> .05; R^2 ^= .00], while both proximity to recreation [F_change _(1,347) = 3.79, *p *< .05; R^2 ^= .01] and conscientiousness [F_change _(1,345) = 3.95, *p *< .05; R^2 ^= .01] displayed moderator effects on the intention-walking relationship. Further, when these were entered into a regression equation hierarchically, each explained 1% of the variance. Slope analyses for proximity to recreation and conscientiousness are presented in Figures 2 and 3 respectively. Interpreting these effects identified that high recreation proximity resulted in a larger effect of intention on walking (β = .45) than low levels (β = .12), while participants high (+1 SD) and medium (within 1 SD) in conscientiousness resulted in a larger effect of intention on walking (β = .49 & .45 respectively) than low levels (-1 SD; β = .24) of conscientiousness.

**Table 4:** Perceived environment, planning, and personality as moderators of intention when predicting walking (n = 358).

	F_change_	df	R^2^_change_	β^1^	β^2^
(Block #1)	18.08*	4,340	.18		
intention				.42**	.37**
planning				-.01	-.01
conscientiousness				.00	.03
proximity to recreation				.06	.06
					
(Block #2)	2.95*	3,337	.02		
Planning × intention					-.07
conscientiousness × intention					.10*
proximity to recreation × intention					.11*

Note: *p < .05; ** *p *< .01. *β*^1–2 ^= standardized regression coefficients for equations #1, and #2. df = degrees of freedom.

## Discussion

This study was the first to attempt an integration of social cognitive, perceived environment, and personality factors to predict leisure-time walking behaviour. Overall, the results complimented prior findings in each of these domains while extending the existing literature on walking.

As hypothesized, the perceived environment, but not personality, was associated with walking. Specifically, close proximity to retail infrastructure, quality of the walking infrastructure, and the aesthetics of the neighbourhood were correlates of walking. These findings parallel general environmental and PA research [18-22,64]. The results for proximity to retail and neighbourhood aesthetics also replicate prior work focused in British Columbia [11] and the addition of quality walking infrastructure may represent the larger variability in the sampling frame (i.e., from City of Victoria to the entire province of British Columbia). Also similar to prior findings with environmental variables, the effect sizes are in the small range [64]. Small effect sizes are likely important to public health initiatives [65], thus it may be prudent for community planners to consider these factors during neighbourhood design and revitalization projects.

The null finding for an extraversion-walking relationship replicates prior work [30], but the current study extends this finding to conscientiousness and neuroticism in a population sample. The indication that walking is unrelated to personality should be considered a positive, because its impact on human behaviour may be fundamentally basic/endogenous and difficult to intervene upon [26].

Our main analysis integrated the environmental characteristics salient to walking within a TPB model that also included a planning construct. Overall, this integrated model explained 25% of the variance in walking which is similar to basic TPB and PA [8] and prior walking research [11,12]. Of key interest, perceived proximity to retail predicted walking independent of the TPB. This result was different from the full mediation of this variable found in the only other study to apply the TPB [11], but small independent effects of the perceived environment on PA are common in existing social cognitive and perceived environmental integration research [23-25,66]. The results suggest that participants who live closer to retail may end-up walking more than originally intended. From a theoretical perspective, the result does not completely support the mediation tenet of variables "external" to TPB structure. Indeed, the finding supports a recent model suggested by Fishbein [67] whereby the environment may affect behaviour independent of initial intention. The hypothesis that some PA is incidental, and dependent upon ones environment, is also a fundamental tenet of social-ecological models [15]. These results, in concert with most prior work, provide support for this theorizing.

Still, the largest predictor of leisure-time walking was one's intention to walk. Thus, walking is primarily a motivation-based behaviour. In turn, walking intention was predicted by affective and instrumental attitudes and PBC, and the effects of walking infrastructure quality and aesthetics were subsequently mediated by attitudes. The four studies that have applied the TPB to understanding walking all differ in their relative contributions from attitude, subjective norm, and PBC constructs [11-13]. Most notable, differences appear to be in the attitude construct, where two studies have shown attitudes as predictors of walking intent/behaviour (present study and [11]) and two studies have not [12,13]. This is probably due to measurement differences in the definition of walking (i.e., sustained leisure-time walking compared to total walking). Total walking may be largely incidental to ones appraisal of the behaviour because it is fundamental to mobility of any kind and for multiple purposes. By contrast, sustained walking during one's leisure-time would seem more dependent upon the appraisal of the behaviour itself. Future research is needed to test this conjecture.

Another interesting finding in this integrated model was the null effect of planning on walking. Planning has had relatively consistent support in the health behaviour literature as a construct that either augments or even mediates intention-behaviour relations [31]. This was the first study to apply the planning construct to walking within a TPB framework, but our findings are almost an exact replication of Rhodes et al. [34]. Three factors may be contributing to this result. First, planning may not be particularly important to walking independent of motivation itself. Walking is noted for its ease to physically perform, access, and low cost. Perhaps planning is not as essential to regular walking because one does not need to overcome these barriers. Some evidence of this theorizing is also present from the null effect of PBC on walking independent of intention. Second, from a methodological perspective, intention and planning may be too collinear to produce unique contributions from each. In these data, intention and planning correlated r = .76, despite attempts to separate their measurement domains. Thus, although distinctions between the two constructs can be made theoretically, participants may not have drawn the same distinctions when responding to the items. Third, planning may be more critical for the initial behaviour change process and not a general construct within the TPB model. A vast majority of the population are in PA stasis (i.e., not changing their PA over short periods of time) [68], and this may attenuate the effect that planning has on those who are actually changing their walking behaviour. Future research is needed to test these possibilities.

A second purpose of this study was to evaluate planning, conscientiousness, and proximity to recreation as moderators of the intention-behaviour relationship. All of these variables have been shown to moderate this relationship in prior work [11,33,38], but they have not been combined to partial-out potential redundancies or to create an integrated model. As hypothesized, conscientiousness and proximity to recreation both moderated the intention-walking relationship. The overall size of this effect was modest (i.e., 2% variance explained) but interactions in survey designs are often difficult to identify due to limited range in the extreme cells [69]. Thus this effect should be considered meaningful. The effect of close proximity to recreation facilities and parks on the intention-walking relationship suggests that those individuals who live closer to recreation have an easier time translating intentions into action. This may be because close proximity improves the ease of acting on one's intentions or because it cues people to follow through with their initial motives. Regardless, the result may have a practical application for regional and community planners: it appears increasing recreation land-mix may help close the intention-walking gap.

For conscientiousness, less conscientious individuals showed a lower intention-behaviour relationship than their moderate and high conscientiousness counterparts. This makes sense, as conscientious people are considered dutiful, achievement-oriented and orderly; following through with one's intentions seems a logical course of action for conscientious people. What was surprising, however, is that planning did not moderate this intention-walking relationship. Although this null finding has been reported before [34], planning has been cited as the potential mechanism, and even a possible intervention for the conscientiousness interaction with intention and behaviour [37,38]. This null finding, therefore, does not support prior theoretical conjecture. Although experimental testing is needed, it may be that conscientiousness affects the intention-behaviour gap on a basic motivational (e.g., achievement striving) rather than an instrumental (e.g., organization, planning) level.

This study needs to be interpreted within the context of its limitations. First, the sampling frame of British Columbia (BC) may not generalize to other regions. BC is the most active province in Canada [4] and its two major cities feature mild climates. Second, although the sample obtained for this research was representative of the BC adult population in terms of sociodemographics [41] and PA [4], the baseline survey response rate was modest and the subsequent attrition rate was high. If differences in terms of PA cognitions and behaviour exist between those who completed the questionnaire and those who did not, it will bias our results. Finally, the walking measure was self-report which can introduce measurement error, particularly with lower intensity activities like walking. Future replication research using objective measures (e.g., pedometry, accelerometry) would be desirable.

## Conclusion

In summary, a model that integrated the TPB, perceived environment, personality, and planning provided evidence that leisure-time walking is largely an intention-based behaviour with an additional independent contribution from one's proximity to neighbourhood retail shops. Intention, in turn, may be predicated on attitudes about walking and PBC, and perceived neighbourhood aesthetics and walking infrastructure may affect walking behaviour through attitudes. The intention-walking relationship, however, may be moderated by conscientiousness and proximity to neighbourhood recreation facilities. Overall, walking behaviour may be affected by environmental, social cognitive, and endogenous individual differences that need to be addressed in intervention efforts.

## Competing interests

The author(s) declare that they have no competing interests.

## Authors' contributions

RER conceived of the study, crafted the design, coordinated data collection, preformed the analyses, and drafted the manuscript. CMB contributed to the analyses and drafting of the manuscript. Similarly, KSC and RCP participated in the design of the study and helped draft the final manuscript.
